# *In vitro* mutagenicity assay (Ames test) and phytochemical characterization of seeds oil of *Helianthus annuus* Linné (sunflower)

**DOI:** 10.1016/j.toxrep.2016.09.006

**Published:** 2016-09-13

**Authors:** Nelma de Mello Silva Oliveira, Marielly Reis Resende, Daniel Alexandre Morales, Gisela de ragão Umbuzeiro, Marcelo Fabiano Gomes Boriollo

**Affiliations:** a Laboratório de Ecotoxicologia e Microbiologia Ambiental, Faculdade de Tecnologia, Universidade Estadual de Campinas (FT/UNICAMP), Limeira, SP, Brazil; b Laboratório de Farmacogenética e Biologia Molecular, Faculdade de Ciências Médicas, Universidade José do Rosário Vellano (UNIFENAS), Alfenas, MG, Brazil; c Centro de Pesquisa e Pós-graduação em Ciência Animal, Área de Patologia e Farmacologia Animal, Universidade José do Rosário Vellano (UNIFENAS), Alfenas, MG, Brazil; d Laboratório de Microbiologia e Imunologia & Laboratório de Genética Molecular, Departamento de Diagnóstico Oral, Faculdade de Odontologia de Piracicaba, Universidade Estadual de Campinas (FOP/UNICAMP), Piracicaba, SP, Brazil

**Keywords:** α-Linolenic Acid (PubChem CID: 5280934), Arachidic Acid (PubChem CID: 10467), Behenic Acid (PubChem CID: 8215), Lignoceric Acid (PubChem CID: 11197), Linoleic Acid (PubChem CID: 5280450), Myristic Acid (PubChem CID: 11005), Oleic Acid (PubChem CID: 445639), Palmitic Acid (PubChem CID: 985), Palmitoleic Acid (PubChem CID: 445638), Stearic Acid (PubChem CID: 5281), *Helianthus annuus* L., Sunflower oil, Genetic toxicity, Gas chromatography

## Abstract

The objective of this research was to investigate the genotoxic potential of the oil of *H. annuus* L. (sunflower) seeds via the Ames test as well as its oxidative properties and lipid composition. The pre-incubation method, system metabolic activation (S9 fraction) and five *S. typhimurium* strains (TA97, TA98, TA100, TA1535 and TA102) were employed for the Ames test. The oxidative stability and fatty acid composition were analyzed by standard methods and gas chromatography. A revertant analysis showed no significant differences between the treatment doses (10–200 μl/plate) and the negative controls, regardless of S9^+^ and S9^−^, and included all of the *S. typhimurium* strains. Chromatographic analysis showed high levels of polyunsaturated fatty acids, followed by monounsaturated, saturated and total trans-isomers. Among the polyunsaturated, monounsaturated and saturated fatty acids, linoleic, oleic and palmitic acids predominated. The results suggest that the sunflower oil is not genotoxic as indicated by frameshift mutations and base pair substitutions regardless of the treatment dose, but shows dose-dependent toxicity. The oxidative properties of the sunflower oil were consistent with the requirements of national and international standards. However, its composition could also indicate phytotherapeutic properties.

## 1 Introduction

The cultivated sunflower (*Helianthus annuus* L.) is one of the 67 species of the genus Helianthus and is a dicotyledonous plant and member of the Compositae (Asteraceae) family, having a typical composite flower [1]. Sunflower seed oils are high in saturated fatty acids (lauric acid C12:0, maristic C14:0 palmitic C16:0 and stearic C18:0), monounsaturated fatty acids (oleic acid C18:1, n-9), and poly-unsaturated fatty acids (linoleic acid C18:2, n-6, and α-linolenic C18:3, n-3) [2]. The unsaturated fatty acids are the most abundant, especially linoleic acid. In turn, linoleic acid makes vegetables oil more susceptible to lipid oxidation [3] and therefore favors the formation of substances (e.g., peroxides, hydroperoxides and free radicals) that cause spoilage [4] in addition to the genotoxicity caused by the reaction between these substances and DNA molecules [5]. In addition, linoleic acid is a precursor of arachidonic acid [6], which participates in the synthesis of biologically active mediators, such as prostaglandins, thromboxanes and leukotrienes. These substances act as inflammatory mediators [7], [8], stimulating local neovascularization, cell migration, proliferation and the differentiation of fibroblasts, along with extracellular matrix synthesis that acts directly on healing [9], [10].

Sunflower seed oil has potential phytotherapeutic properties [10], and some research also supports the phytotherapeutic effectiveness of the oil and an aqueous or alcoholic extract of sunflower seeds for the relief of asthmatic symptoms and other diseases [11], gastric protection [12], [13], healing properties [14], anti-inflammatory action [15], [16], [17] and antimicrobial properties [12], [14], [18], [19]. In addition, a limited number of investigations that investigated the genotoxic action of various oils, including sunflower seed oil, have gone unnoticed. For example, vegetable oils for human consumption showed high (linseed oil) and weak (sesame oils, wheat germ and soybean) genotoxic responses or even the absence of genotoxicity (sunflower oil, olive oil and refined olive oil extra-virgin) according to a mutation and somatic recombination test (SMART) in *Drosophila melanogaster* [20]. In another study, lymphocytes incubated with an aqueous extract of sunflower oil submitted to thermal stress exhibited high rates of chromosomal breakage and were significantly different from those of lymphocytes incubated with the same concentrations of the aqueous extract of sunflower oil in the absence of heat. Furthermore, in tests with HepG2 or HUVEC cells, sunflower oil subjected to heat stress was clastogenic and showed dose-dependent cytotoxicity [21]. The absence of clastogenicity and/or aneugenicity in two sources of oil and a tincture of *H. annuus* L. (sunflower) seeds was also confirmed by in vivo micronucleus assays in mouse bone marrow and was dose-independent, time-independent and sex-independent, except for the oil. However, systemic toxicity of sunflower oil might be dependent on its origin and dose [22].

Thus, further studies on the genotoxicity of sunflower extracts and oils (seeds, flowers and leaves) must be conducted to determine their effects and potential genotoxic mechanisms, especially for setting limits for human use. The Ames test (Salmonella/Microsome test) has been employed as an indicator of the carcinogenic potential in mammals and uses bacterial strains of *S. typhimurium* that are auxotrophic for histidine (*his*^−^) (i.e., are unable to grow in a minimal culture medium without histidine) because of the presence of mutations in the histidine operon. These strains are used to detect gene mutations, base pair substitutions and frameshift types. However, revertant colonies (i.e., histidine prototrophs) can be quantified after exposure to test substances in the presence or absence of an exogenous metabolic activation system, which indicates the occurrence of gene mutations by the restoration of bacterial metabolism and growth in minimal culture medium [23], [24], [25]. Thus, this *in vitro* assay can be used in the screening of new chemicals and drugs as well as to provide a high predictivity of carcinogenicity due to mutagenesis [24]. Some vegetable oils (*Ocimum selloi* [26], *Melaleuca alternifolia* and *Lavandula angustifólia* [27], *Azadirachta indica* [28], *Curcuma longa* L. [29]) have been previously evaluated by the Ames test.

To contribute to the information on the genotoxic potential of vegetable oils, this study evaluated the mutagenic effects of the pharmaceutical oil of *H. annuus* L. seeds (sunflower) in the Ames test using *S. typhimurium* strains TA97a, TA98, TA100, TA102 and TA1535. The oxidative properties and lipid composition of this oil were also assayed in oxidative stability tests (iodine, peroxide and acidity index) and by gas chromatography (GC).

## 2 Material and methods

### 2.1 Phytotherapeutic sunflower oil

The pharmaceutical oil of *H. annuus* L. (sunflower) seeds (CAS # 8001-21-6) was purchased commercially and stored according to the manufacturer's recommendations (Fagron Farmacêutica do Brasil, São Paulo, SP, Brazil, lot 14010155B: relative density equal to 0.923 g/cm^3^, iodine index equal to 126 g/100 g, acidity index equal to 0.03% and peroxide index equal to 0.03 mequiv. O_2_/kg).

### 2.2 Ames test (Salmonella/Microsome test)

Bacterial strains of *S. typhimurium* TA97a, TA98, TA100, TA102 and TA1535 were kindly provided by Companhia Ambiental do Estado de São Paulo (CETESB, SP, Brazil) to Laboratório de Ecotoxicologia e Microbiologia Ambiental of Prof. Dr. Abílio Lopes – LEAL, stored and maintained as provided in the standard protocol [25]. The test was performed with the pre-incubation method according Mortelmans and Zeiger (2000) [24] and Guideline for testing of chemicals [25]. Initially, each bacterial strain was grown in 20 ml of nutrient broth (Nutrient Broth Oxoide no. 2, code # CM0067, Thermo Fisher Scientific Inc.) at 37 °C for 16 h (overnight) under constant shaking at 160–170 r.p.m (Incubator with Orbital Agitation Platform – Shaker model 430, Nova Ética, Vargem Grande Paulista, SP, Brazil). Then, 100-μl aliquots of each freshly grown bacterial culture (1–2×10^9^ CFU/ml) were added to assay tubes containing (*i*) a known volume of sunflower oil (10, 20, 50, 100 and 200 μl/plate) and 500 μl of the S9 mixture [phosphate buffer, NADPH glucose-6-phosphate, solution of salts (MgCl_2_ and KCl), and the S9 fraction (S9 microsomal fraction of homogenized rat liver: post-mitochondrial fraction supplemented with a cofactor, prepared from the liver of rodents treated with an agent enzyme inducer, arocloror 1254, MOLTOX^®^, Molecular Toxicology, USA)] – system with metabolic activation – or (*ii*) known volumes of sunflower oil (10, 20, 50, 100 e 200 μl/plate) and 500 μl of phosphate buffer (Na_2_HPO_4_ and NaH_2_PO_4_·H_2_O) – system without metabolic activation –, and pre-incubated at 37 °C for 30 min.

Prior to testing, aliquots of the S9 fraction were prepared according to the manufacturer's specifications and stored in 2-ml sterile Eppendorf-type tubes at −20 °C. The reagent 2-Aminoanthracene (2.5 μg/plate; CAS Number 613-13-8, Cat. #A38800 Aldrich, Sigma-Aldrich Chemical Co.) was used as a positive control in analysis systems with metabolic activation of all of the *S. typhimurium* strains. For analysis systems without metabolic activation and as a positive control, 4-Nitroquinoline *N*-oxide (0.5 μg/plate; CAS Number 56-57-5, Cat. #N8141 Aldrich, Sigma-Aldrich Chemical Co.) was used in the assays with the TA97a, TA98 and TA100 strains and sodium azide (5 μg/plate; CAS Number 26628-22-8, Cat. #V000494 Vetec, Sigma-Aldrich Chemical Co.) and hydrogen peroxide (50 μg/plate; CAS Number 7722-84-1, Cat. #H1009 Sigma, Sigma-Aldrich Chemical Co.) were employed in the assays with strains TA1535 and TA102, respectively. Phosphate buffer was used as a negative control of analysis systems with and without metabolic activation with all *S. typhimurium* strains [24], [25].

After the pre-incubation period, 2 ml of surface agar (top agar) adjusted to 45 °C [10.3 mM NaCl, 0.5 mM biotidine solution (histidine and biotin) and bacteriological agar 0.6% (w/vol)] were added to each test tube, vortexed for 30 s and dispensed on Petri dishes (90 mm × 150 mm) containing 20 ml of minimal Vogel Bonner medium [20 ml of 50 × (10 g of MgSO_4_·7H_2_O, 100 g of C_6_H_8_O_7_·H_2_O, 500 g of K_2_HPO_4_, 175 g of NaNH_4_HPO_4_·4H_2_O, 1000 ml of H_2_O type 1 q.s.p.); 200 ml of glucose solution 10% (w/vol); 780 ml of bacteriological agar 1.92% (w/vol)] for the TA98, TA100, TA102 and TA1535 strains, and minimal agar with added glucose at 0.4% (w/vol) for strain TA97a. These plates were kept at room temperature until the complete solidification of the top agar. Revertants (*his*^+^) were counted after incubation at 37 °C for 66 h [24] and Guideline for testing of chemicals [25].

### 2.3 Identity standard and quality and lipid characterization of oil

The sunflower oil was characterized by chemical analysis of its oxidative stability, such as the acidity index (mg KOH/g) Iodine (g/100 g oil) and peroxide (mequiv. O_2_/kg) according to standard methods [30]. These analyses provide information about the identity and quality of the oil after processing and marketing. The fatty acid profile was determined as previously described [30], [31], [32], [33] employing a Perkin Elmer Clarus 500 gas chromatograph (Norwalk, CT) with an automatic injector and flame ionization detector. Conditions: A 50 m × 0.25 mm WCOT fused silica column (CAPILLARY CP-Sil 88, CP6173, Agilent Technologies, CA, USA), with the injector at 270 °C and a flame ionization detector at 310 °C. Initial oven temperature was 140 °C for 2 min and was increased in 2 °C increments for 60 s to 235 °C, held at 235 °C for 10 min, and finally raised to 270 °C. Hydrogen was used as the carrier gas at a flow of 30 ml per min. Individual fatty acids were identified by comparing their retention times with those of purified standards (fatty acid esters). The peak area was calculated using the chromatographic integrator and chromatography software and expressed as a relative percentage of each fatty acid in relation to the total fatty acids.

### 2.4 Statistical analysis

The Ames test data were subjected to one–way analysis of variance (ANOVA) and medium comparison with Scott-Knott’s test (α = 0.05) [34] using the SISVAR computer software [35]. Also the mutagenic ratio was calculated according to the following equation: $RM=\frac{\bar{x_{1}}}{\bar{x_{2}}}$, where *RM*, $\bar{x_{1}}$e $\bar{x_{2}}$ correspond to the mutagenicity ratio, the mean number of revertant colonies on the test plate and the mean number of revertant colonies on the negative control plate, respectively. A compound is considered to be genotoxic when (*i*) *RM* ≥ 2 was noted in at least one dose tested and (*ii*) ≥1 strain showed a significant dose-response (*p* < 0.05) among the tested concentrations or spontaneous revertants (negative control) [36], [37].

## 3 Results and discussion

### 3.1 Mutagenicity of sunflower oil

This study indicated that there was no significant increase (*p* < 0.05) in the number of revertant colonies of the *S. typhimirium* strains TA97a, TA98, TA100, TA102 and TA1535 for any of the tested doses of sunflower oil (10–200 μl/plate) compared to the negative controls with and without exogenous metabolic activation system (Table 1). Also any dose tested of the sunflower oil (10–200 μl/plate) presented *RM* > 2 (data not shown).

**Table 1:** Mean number of revertant colonies (TA97a, TA98, TA100, TA102 and TA1535) observed on experimental treatment with sunflower oil including the reference mutagens and the negative control in the Ames test (pre-incubation).

Dose (μl/plate)		TA97a	TA98	TA100	TA102	TA1535
*S9* (−)						
Negative control		144.67 ± 5.86	27.33 ± 5.03	180.00 ± 27.00	463.33 ± 43.25	27.00 ± 1.73
10	Sunflower oil	145.67 ± 8.33	27.33 ± 8.08	172.33 ± 36.12	421.00 ± 53.7	21.67 ± 2.31
20		151.33 ± 24.01	28.00 ± 10.58	125.33 ± 13.05^a^	462.67 ± 51.39	21.33 ± 2.08
50		126.50 ± 4.95	29.33 ± 8.96	124.00 ± 20.88^a^	456.67 ± 35.57	22.00 ± 5.57
100		129.00 ± 8.54	31.00 ± 10.82	132.00 ± 5.29^a^	477.67 ± 55.97	19.67 ± 4.93
200		125.00 ± 6.56	25.67 ± 49.93	107.00 ± 18.36^a^	469.00 ± 18.00	20.67 ± 0.58
Positive control^1^		1025 ± 102.40^a^	426.00 ± 33.31^a^	4061.00 ± 547.85^a^	892.00 ± 181^a^	2707.00 ± 99.71^a^

*S9* (+)						
Negative control		190.67 ± 13.01	38.33 ± 4.73	185.67 ± 33.38	611.00 ± 28.28	16.67 ± 4.51
10	Sunflower oil	155.00 ± 0.58	37.33 ± 7.64	147.67 ± 14.05	608.50 ± 3.54	16.33 ± 1.15
20		168.33 ± 5.51	25.33 ± 5.86^a^	166.67 ± 3.51	610.33 ± 18.15	17.33 ± 0.58
50		178.00 ± 16.52	22.00 ± 3.46^a^	152.67 ± 21.08	519.67 ± 29.02^a^	14.00 ± 3.61
100		165.67 ± 16.50	15.00 ± 1.73^a^	153.67 ± 16.62	475.67 ± 30.75^a^	14.00 ± 2.00
200		137.67 ± 24.01^a^	22.33 ± 2.89^a^	137.33 ± 19.66	479.67 ± 90.75^a^	17.00 ± 6.56
Positive control^2^		3200 ± 202.23^a^	1510.33 ± 222.46^a^	7932.66 ± 267.4^a^	912.66 ± 33.50^a^	244.66 ± 26.63^a^

CV (%)		9.14	17.97	12.80	7.69	23.27

The numbers indicate the means and standards deviation values of CFU in triplicate assay systems.

CV = coefficient of variation.

Without (−) and with (+) S9 microsomal fraction of homogenized rat liver (post-mitochondrial fraction supplemented with a cofactor, prepared from the liver of rodents treated with an enzyme inducer agent arocloror 1254, MOLTOX^®^, Molecular Toxicology, USA).

Negative control: phosphate buffer.

Positive control^1^: 4-Nitroquinoline N-oxide (CAS Number 56-57-5, Cat. #N8141 Aldrich, Sigma-Aldrich Chemical Co.) for TA97a, TA98 and TA100; Sodium azide (CAS Number 26628-22-8, Cat. #V000494 Vetec, Sigma-Aldrich Chemical Co.) for TA1535; Hydrogen peroxide (CAS Number 7722-84-1, Cat. #H1009 Sigma, Sigma-Aldrich Chemical Co.) for TA102.

Positive control^2^: 2-Aminoanthracene (CAS Number 613-13-8, Cat. #A38800 Aldrich, Sigma-Aldrich Chemical Co.).

a Significantly different from the corresponding negative control values (ANOVA and Scott-Knott test, *p* < 0.05).

The Ames test has been previously used for mutagenicity evaluation of some vegetable oils. For example, *Ocimum selloi* oil was analyzed at concentrations of 500–700 mg/plate using *S. typhimurium* strains TA 97a, TA98 e TA100 in the presence or absence of metabolic activation (S9 fraction) [26]. The essential oils of *Melaleuca alternifolia* and *Lavandula angustifolia* were also analyzed at concentrations of 0.28, 0.88 and 2 mg/plate with *S. typhimurium* strains TA98 and TA100 and *E. coli* WP2 *uvr*A, respectively, in the presence or absence of metabolic activation (S9 fraction) [27]. Neem oil was studied at concentrations of 0.01–10 mg/plate using *S. typhimurium* strains TA98, TA100, TA102, TA1535 and TA1537, as well as at concentrations of 0.1–100 μg/plate, diluted in DMSO, using strains TA98, TA100 and TA102 in the presence or absence of metabolic activation (S9 fraction) [28]. The essential oil of *Curcuma longa* L. was analyzed at concentrations of 0.1–3 mg/plate with *S. typhimurium* strains TA98, TA100, TA102 and TA1535 in the presence or absence of metabolic activation (S9 fraction) [29]. All of these Ames test results showed that these oils were not genotoxic regarding frameshift mutations (TA97, TA98 and TA1537) and base pair substitutions (TA100, TA102, TA1535 and *E. coli* WP2 *uvr*A) [24].

Recently, a micronucleus test in mouse bone marrow showed no clastogenic and/or aneugenic effects of the tincture and *H. annuus* L. seed (sunflower) oils from two sources regardless of the dose (0.25–2 g/kg) and treatment time (24 and 48 h), but the results were sex-independent (sunflower tincture) or sex-dependent (sunflower oils) [22]. Differently, in lymphocytes incubated with a water extract of heated sunflower oil containing 0.075 or 0.15 μM thiobarbituric acid-reactive substances (this extract had a high content of polar aldehydes), the rate of chromosomal breakage was 18.4% and 23.1% compared to 8.7% and 6.6% or 8.1% and 9.2%, respectively, in lymphocytes incubated with the same volume of a water extract from a non-heated oil or distilled water [21]. In HepG2 or HUVEC cells, the cytotoxic properties of heated sunflower oil were found to be dose dependent, and cytotoxicity occurred at concentrations as low as 0.25 μM. In contrast, the same volume of non-heated oil or distilled water was non–toxic in these cells [21]. These results showed that the water extract obtained from heated oil is clastogenic and, in higher doses, cytotoxic. These data also suggested that the water extract obtained from heat-stressed cooking oils that had a high aldehyde content was clastogenic. It was speculated that the ingestion of large amounts of these products could impact human health, especially for diseases resulting from chromosomal breakage, such as certain congenital malformations and certain types of cancer. This last fact can be corroborated by previous reports indicating that the administration of heat-stressed sunflower oil to rats is teratogenic [21].

In this study, a significantly lower number (*p* < 0.05) of revertant colonies was observed in sunflower oil treatments with strain TA100 (20–200 μl/plate S9^−^), as well as strains TA97a (200 μl/plate S9^+^), TA98 (20–200 μl/plate S9^+^) and TA102 (50–200 μl/plate S9^+^), compared with their negative controls (Table 1), suggesting that the dose-dependent toxicity of sunflower oil started from 200 μl/plate for *S. typhimurium* strain TA97a, 50 μl/plate for TA102 and 20 μl/plate for TA98 and TA100 under the conditions tested. The analysis obtained from the PCE/NCE ratio (PCE: polychromatic erythrocyte; NCE: normochromatic erythrocyte) during the micronucleus assay in mouse bone marrow also suggested that the systemic toxicity of sunflower oil might be source-dependent on the highest dose used [22]. However, the Ames test indicated no mutagenic responses of sunflower oil at the tested conditions: (10 μl/plate S9^+^: TA98; 10–20 μl/plate S9^+^: TA102; 10–100 μl/plate S9^+^: TA97a; 10–200 μl/plate S9^+^: TA100 and TA1535; 10 μl/plate S9^−^: TA100; 10–200 μl/plate S9^−^: TA97a, TA98, TA102 and TA1535). These results could be explained by the Brazlian standards of the identity and quality of sunflower oil, which are established by ANVISA [38]. This norm requires the verification of the identity and minimum quality of vegetable oils, vegetable fats and vegetable creams. The specific requirements are an acidity in refined oils and fats equal to 0.6 mg KOH/g at most and a peroxide index equal to 10 mequiv. O_2_/kg at most in refined oils and fats. These standards are in accordance with the international standards proposed by the Codex Alimentarius [39], which established the same standards for acidity and peroxides.

### 3.2 Identity, quality and lipid characterization of sunflower oil

The chemical analyses of the oxidative stability for the characterization of sunflower seed oil showed an acidity index equal to 0.16 mg KOH/g, iodine equal to 124 g/100 g, and oil and peroxide equal to 6.23 mequiv. O_2_/kg. According to the RDC 270 [38] and the Codex Alimentarius [39] the determinations are within the nationally and internationally required standards.

The identity and quality parameters evaluated in this study were compared with the results previously reported for *Citrullus colocynthis* (L.) Schrad seed oil and *Helianthus annuus* (sunflower) seed oil [40]. In this study, the peroxide value (ISO 3960), acidity (the percentage of free fatty acids was calculated as oleic acid) (ISO 660) and saponification number (ISO 3657) of the seed oil were determined according to the International Organisation for Standardisation (ISO) standards. The seed oils showed the following properties: acid value of 3.14 ± 0.11 (*C. colocynthis*) and 2.80 ± 0.08 (*H. annuus*); free fatty acid – FFA (as oleic%) of 1.57 ± 0.11 (*C. colocynthis*) and 1.40 ± 0.08 (*H. annuus*); saponification value (mg KOH/g) of 204.63 ± 0.73 (*C. colocynthis*) and 197.45 ± 0.68 (*H. annuus*); iodine number (g/100 g oil) of 123.31 ± 1.32 (*C. colocynthis*) and 118.56 ± 0.98 (*H. annuus*); peroxide value (mequiv. O_2_/kg) of 9.42 ± 0.18 (*C. colocynthis*) and 6.07 ± 0.05 (*H. annuus*). These findings agree with our results for sunflower oil for iodine and peroxide, except for the saponification value, which was significantly lower. It also suggests that the sunflower oil tested for genotoxicity had characteristics that made it less susceptible to lipid oxidation and therefore more resistant to rancidity, which potentially ensured the integrity and preservation of the sunflower oil without interfering with the levels of liposoluble vitamins and essential fatty acids.

The FFA content of both oils (*C. colocynthis* and *H. annuus*) was low and found to be well correlated with the moisture values (7.51% and 3.75%, respectively) because FFAs are the result of the hydrolysis of the oil. Furthermore, it is well known that free fatty acids are more susceptible to lipid oxidation, which can explain their relatively high peroxide value (9.42 mequiv. O_2_/kg) of *C. colocynthis* seed oil compared to sunflower oil (6.07 mequiv. O_2_/kg) [40]. In addition, the low peroxide and FFA values and the absence of a disagreeable flavor and odor in *C. colocynthis* seed oil indicated that this seed is not susceptible to oxidation when intact [41] and can be stored for a long period of time without deterioration until further use in both the food and oleo-chemical industry. *C. colocynthis* seed oil had higher saponification and iodine values than sunflower seed oil [40]. A high saponification value is associated with the presence of shorter chain fatty acids, such as palmitic (C16) or stearic (C18) acids [42]. However, the high iodine value indicated that the oil is rich in double bonds [41]. Unsaturated fatty acids tend to be more reactive toward atmospheric oxygen and undergo oxidation. Consequently, the oxidation of an oil can result in changes that affects its integrity and security, such as the generation of potentially toxic polymeric compounds (e.g., peroxides) [40].

**Fig. 1:**
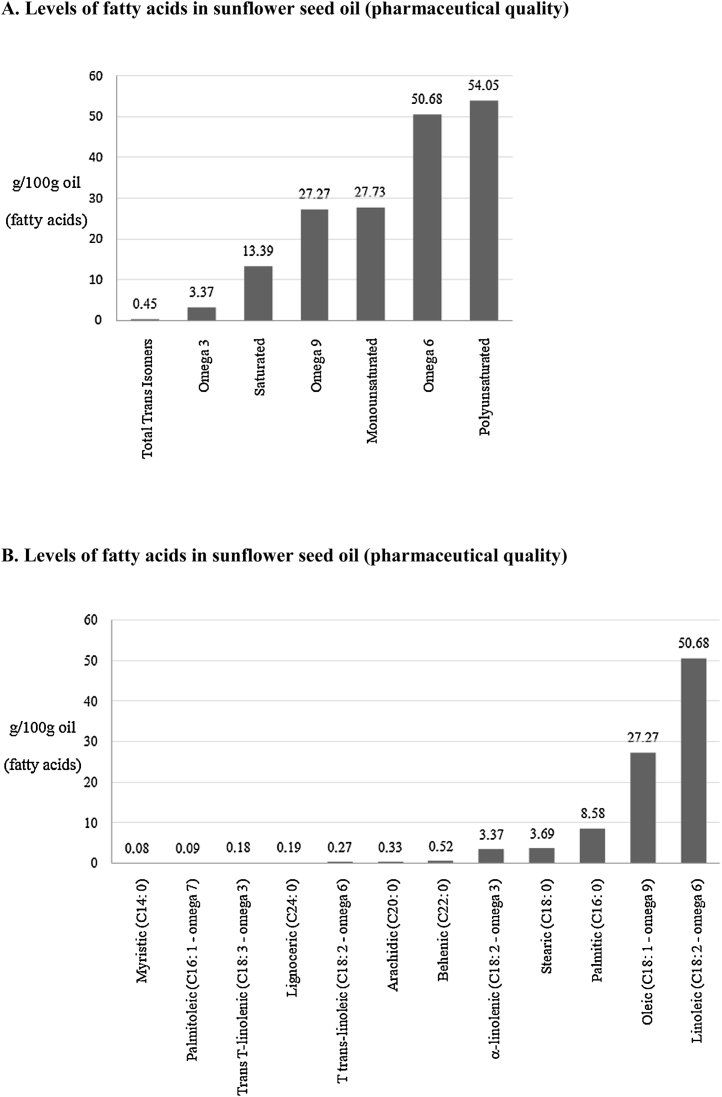
Gas chromatograph (GC) for pharmaceutical oil of *H. annuus* L. (sunflower) seeds (CAS #8001-21-6). Fatty acids profile: (A) polyunsaturated (total, omega 6 and omega 3), monounsaturated (total and omega 9) saturated and total trans-isomers; (B) saturated fatty acids (palmitic, stearic, behenic, arachidic, lignoceric and myristic acids), monounsaturated fatty acids (oleic and palmitoleic acids), polyunsaturated fatty acids (linoleic and α-linolenic acids), and trans-isomers (trans T-linoleic and trans T-linolenic acids).

The lipid characterization of sunflower seed oil revealed that a specific invariant profile was usually found [43], [44], [45], [46]. Initially, our chromatographic analysis (GC) showed a fatty acid content consisting mainly of poly-unsaturated chains (54.05 g/100 g), such as omega 6 (50.68 g/100 g) and omega 3 (3.37 g/100 g), and monounsaturated (27.73 g/100 g), such as omega 9 (27.27 g/100 g), and saturated (13.39 g/100 g) and total trans-isomers (0.45 g/100 g) (Fig. 1A, Table 2), suggesting that it originated from a sunflower species that had not been genetically modified [3]. Among the saturated fatty acids, palmitic acid (C16:0 → 8.58 g/100 g) predominated, followed by stearic (C18:0 → 3.69 g/100 g), behenic (C22:0 → 0.52 g/100 g), arachidic (C20:0 → 0.33 g/100 g), lignoceric (C24:0 → 0.19 g/100 g) and myristic (C14:0 → 0.8 g/100 g) acids. Among the monounsaturated fatty acids, oleic acid (C18:1 → 27.27 g/100 g) predominated, followed by palmitoleic acid (C16:1 → 0.9 g/100 g), and among the poly-unsaturated fatty acids, linoleic acid (C18:2 → 50.68 g/100 g) predominated, followed by α-linolenic (C18:2 → 3.37 g/100 g), trans T-linoleic (C18:2 → 0.27 g/100 g) and trans T-linolenic acids (C18:3 → 0.18 g/100 g) (Fig. 1B, Table 2).

**Table 2:** Fatty acids profile (polyunsaturated, monounsaturated, saturated and total trans-isomers) of pharmaceutical oil of *H. annuus* L. (sunflower) seeds (CAS #8001-21-6) obtained by gas chromatograph (GC).

Fatty acids	Values (g/100 g)
Polyunsaturated	54.05
Linoleic acid (C18:2) (omega 6)	50.68
α-Linolenic acid (C18:2) (omega 3)	3.37

Monounsaturated	27.73
Oleic acid (C18:1) (omega 9)	27.27
Palmitoleic acid (C16:1)	0.9

Saturated	13.39
Palmitic acid (C16:0)	8.58
Stearic acid (C18:0)	3.69
Behenic acid (C22:0)	0.52
Arachidic acid (C20:0)	0.33
Lignoceric acid (C24:0)	0.19
Myristic acid (C14:0)	0.8

Total trans-isomers	0.45
Trans T-linoleic acid (C18:2)	0.27
Trans T-linolenic acid (C18:3)	0.18

The high amount of linoleic acid (C18:2) present in sunflower seed oil can make it more susceptible to oxidation and consequently cause higher cytotoxicity due to the production of free radicals, which might explain our findings on sunflower oil toxicity using *S. typhimurium* strains TA98 (20–200 μl/plate S9^+^), TA100 (20–200 μl/plate S9^−^) and TA102 (50–200 μl/plate S9^+^). On the other hand, the fatty acid composition also contributes to the phytotherapeutic properties, such as in healing and inflammatory processes [6], [8], [10], [47]. Therefore, any change in the chemical composition due to selective breeding, which is spurred by a worldwide demand for more stable varieties of sunflower oil with a reduced risk of fatty acid oxidation (i.e., ↓ linoleic *versus* ↑ oleic acid) [3], could directly change the phytotherapeutic properties of sunflower oil. The therapeutic properties of some oils are closely related to its constitution. Similar to the oil of *H. annuus* seeds used in this study, the *C. colocynthis* and *H. annuus* oil previously studied [40] had high levels of oleic monounsaturated fatty acids (C18:1 → 14, 20 g/100 g and C18:1 → 37, 73 g/100 g, respectively) and polyunsaturated linoleic acid (C18:2 → 66, 78 g/100 g and C18:2 → 45.49 g/100 g, respectively). Due to the linoleic acid, these oils may have an important role in restoring the structure and function of the permeable barrier of the stratum corneum of the skin [48]. Still, inadequate levels of linoleic acid may result in abnormal barrier functions, such as an increased trans-epidermal water loss [49].

## 4 Conclusion

The present research has contributed to the toxicological profile of sunflower oil (pharmaceutical quality) by presenting the results from a well conducted Ames test. The results obtained from the Ames test (Salmonella/microsome test), which was used to indicate the carcinogenic potential, suggest that there is no dose-independent mutagenicity of sunflower oil but it was observed a dose-dependent cytotoxicity. The oxidative properties of sunflower oil were found to be in accordance with the requirements of national and international standards [i.e., acidity index (mg KOH/g), iodine (g/100 g oil) and peroxide (mequiv. O_2_/kg)]. Gas chromatography (GC) revealed high levels of polyunsaturated fatty acids, followed by monounsaturated, saturated and trans-isomers. Among these, the highest were linoleic (50.68 g/100 g), oleic (27.27 g/100 g) and palmitic acids (8.58 g/100 g). The chemical composition might explain the cytotoxicity of sunflower oil to the strains of *S. typhimurium* used in the Ames test. However, the chemical characteristics of these fatty acids might also contribute to the phytotherapeutic properties of oil seeds of *H. annuus* L. (sunflower).

## Conflict of interest

The authors have declared that there is no conflict of interest.
